# Tuberculosis in Sudan: A Comprehensive Narrative Review of Epidemiological Trends, Diagnosis, Drug Resistance, Challenges, and Data Gaps

**DOI:** 10.1002/hsr2.73010

**Published:** 2026-09-15

**Authors:** Mohammad Azhar Kamal, Hisham N. Altayb, Shima M. Abdulgader, Ehssan Moglad

**Affiliations:** ^1^ Department of Pharmaceutics, College of Pharmacy Prince Sattam bin Abdulaziz University Alkharj Saudi Arabia; ^2^ Department of Biochemistry, Faculty of Sciences King Abdulaziz University Jeddah Saudi Arabia; ^3^ DSI-NRF Centre of Excellence for Biomedical Tuberculosis Research, South African Medical Research Council Centre for Tuberculosis Research, Division of Molecular Biology and Human Genetics, Faculty of Medicine and Health Sciences Stellenbosch University Cape Town South Africa

**Keywords:** Diagnosis, MDR, Prevalence, Sudan, TB

## Abstract

**Background and Aims:**

This narrative review aims to provide a comprehensive overview of tuberculosis (TB) in Sudan, focusing on prevalence, drug resistance patterns, and associated challenges while identifying knowledge gaps in scientific research and data needed for effective management.

**Methods:**

A comprehensive literature search was conducted using PubMed, Scopus, Web of Science, and Google Scholar without language or date restrictions. Thirty‐two studies and TB notification data from 2009 to 2025 were analyzed.

**Results:**

Among 32 studies, 41% (13/32) addressed TB prevalence and 59% (19/32) explored drug resistance. Kassala State reported the highest pulmonary TB prevalence (70%), while Khartoum had 17%. Drug‐resistant TB was more prevalent in Khartoum (41.5%) than Kassala (21.4%). Only 23 studies examined HIV coinfections. TB notifications declined from 209 per 100,000 in 2009 to 10 per 100,000 in 2019 but increased to 50 per 100,000 after the COVID‐19 pandemic. Data were lacking for Sudan's western states. Because of the Sudan war, in 2024, 14,310 cases were reported.

**Conclusion:**

This review underlines the scarcity of TB research in Sudan and highlights the urgent need for an integrated health information system to generate accurate, updated data for timely interventions.

## 1 Introduction

Tuberculosis (TB) is the leading cause of death from a single infectious agent, ranking above HIV/AIDS [1]. Due to the Coronavirus Disease 2019 (COVID‐19) pandemic, a 4.5% increase in the number of people falling ill with TB was reported in 2021, reversing years of a slow decline [1]. Despite being a curable disease, TB remains a major public health concern worldwide, especially in developing countries [1, 2, 3]. Although a quarter of the global population is estimated to have been infected with TB [4], only a minority will go on to develop TB disease, and some will clear the infection [5, 6]. The disease usually affects the lungs (pulmonary TB (PTB)) and can also affect other body sites (extrapulmonary TB (EPTB)).

**Figure 1:**
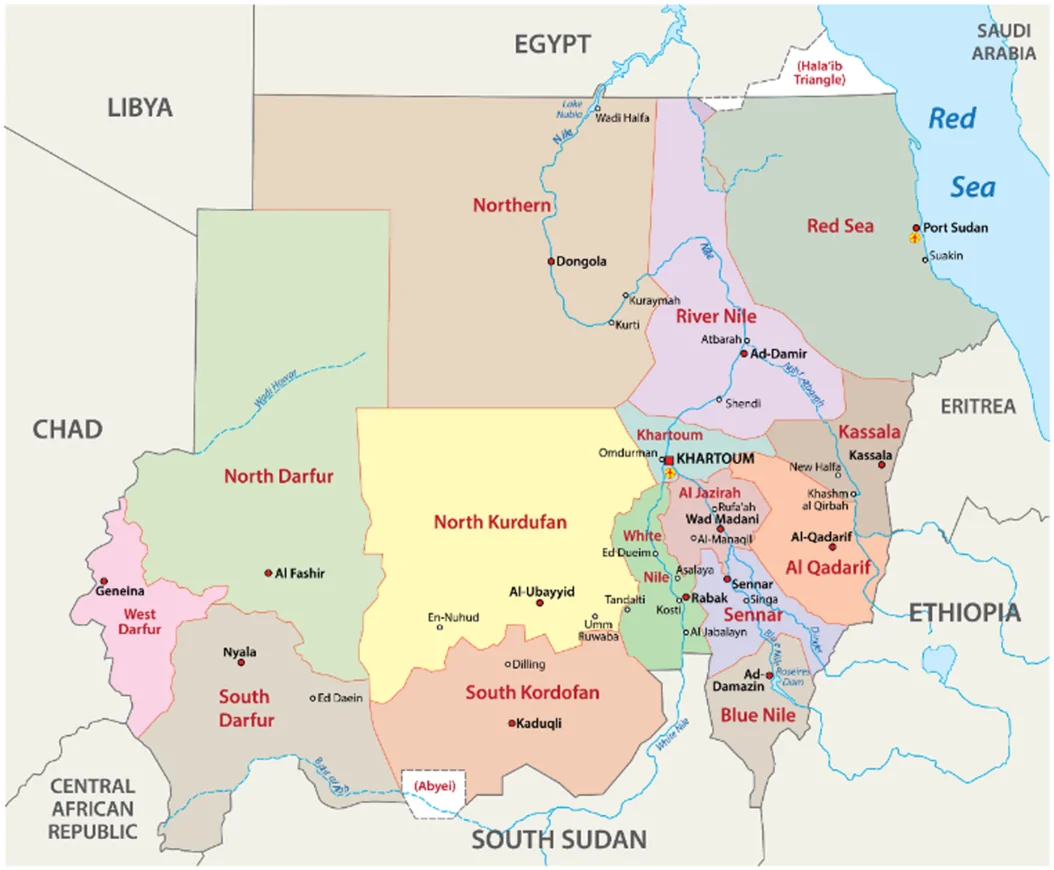
Map of the Republic of Sudan.

Sudan is the 16th largest country in the world, https://www.nationsonline.org/oneworld/egypt.htmwith an area of 1,886,068 km^2^ today, and it has 18 states following the separation from South Sudan in 2011 (Figure 1). Sudan has been affected by armed conflicts and civil wars since its independence in 1956, which resulted in a fragile health system and weak infrastructure. Sudan is considered part of the Eastern Mediterranean WHO Region (EMR), and it carries 8% of the TB burden in the region. The ongoing internal conflicts and civil unrest in Sudan for the past seven decades resulted in a severely malfunctioning governing system with very low (8%) expenditure on health, which complicates the efforts to control communicable diseases such as TB.

The WHO included Sudan for the first time in the national population‐based surveys of the prevalence of TB in 2013 [7]. Nonetheless, there is a limited and scattered body of literature on TB in Sudan; therefore, in this review we aim to discuss the landscape of the disease, highlight the associated challenges and knowledge gaps and, where possible, propose solutions to address these gaps.

## 2 Methods

Through March 2023, we screened PubMed, Scopus, Web of Science, and Google Scholar without language or date restrictions for any studies that reported data on TB in Sudan using the terms “tuberculosis” and “Sudan”; without any language or date restrictions, all age groups are included. *In‐vitro* studies were included only if they reported data on molecular typing or drug resistance. Where possible, and in the absence of research‐generated data, we used secondary data such as TB incidence and case notifications from the WHO Global TB Reports. These reports compile national surveillance data, whereby TB case notifications are reported by the Sudanese National TB Programme (NTP) to the Federal Ministry of Health and subsequently transmitted to WHO through standardized reporting frameworks. An updated literature search was conducted in April 2026 to identify any newly published data.

This review is written according to the Guidelines of proper analysis, reporting, and interpretation of clinical research [8].

### 2.1 Statistical Analysis

Statistical analyses summarized patterns of TB prevalence and drug resistance across the included studies. Descriptive statistics presented resistance rates, prevalence estimates, and distributions across years and geographic regions as proportions and percentages. Temporal trends in multidrug‐resistant TB (MDR‐TB) were evaluated using linear regression analysis. Geographic variation in resistance rates between states was assessed using analysis of variance (ANOVA). Logistic regression analysis examined associations between the presence of resistance‐associated genes and phenotypic drug resistance where appropriate.

All statistical tests were two‐sided, with a priori significance level of *α* = 0.05. *P* values were reported alongside the corresponding data and statistical tests, following standard reporting conventions: *p* < 0.001 for values less than 0.001, values between 0.001 and 0.01 reported to three decimal places, and values of 0.01 or greater reported to two decimal places. Statistical analyses were conducted using R statistical software (version 4.3.1) and relevant packages.

### 2.2 Declaration of Generative AI and AI‐Assisted Technologies in the Manuscript Preparation Process

During the preparation of this manuscript, the authors used ChatGPT Plus for heatmap generation and flowchart preparation. The authors subsequently reviewed and revised the generated content as necessary and take full responsibility for the final content of the manuscript.

## 3 Findings and Discussion

### 3.1 TB Prevalence

Currently, Sudan is in its third year of conflict, and TB remains a serious public health issue as the health‐care system struggles. In 2024, 14,310 cases were reported, but the actual number is likely higher due to under‐reporting in hard‐to‐reach areas. Services are still running in some locations, with 148 TB centers, 8 drug‐resistant TB centers, and 32 molecular labs, but many regions remain without proper surveillance or care [9].

**Figure 2:**
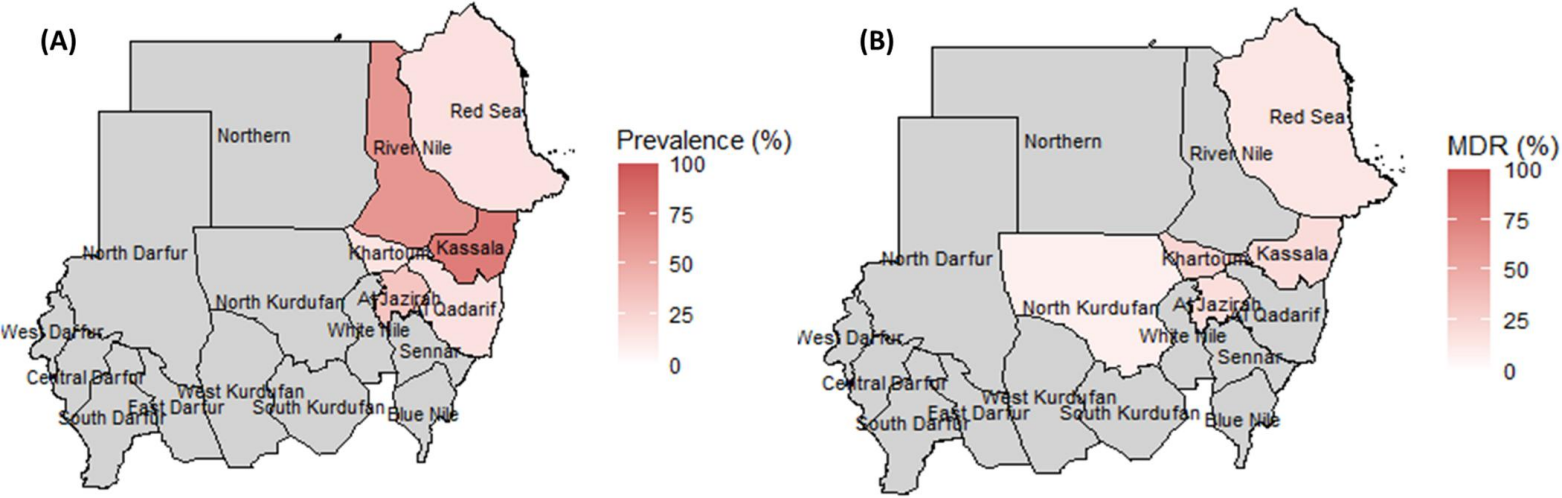
The prevalence of pulmonary TB (A) and multidrug‐resistant MTB (B) in Sudan.

The prevalence of TB in Sudan has been increasing in the past five decades (1967–2014) and is ranked amongst the highest TB burden countries in the EMR [10, 11]. In 2009, the prevalence per 100 000 persons was 209, with an incidence of 50 000 new cases [12, 13], which declined to 6587 cases reported in 2012 [14]. In 2018, a total of 20,638 cases were reported with an estimated national TB incidence of 71 per 100,000 persons [15]. The prevalence of TB varied between the different states, and Kassala state (eastern Sudan) recorded the highest TB notifications, reaching 275 per 100,000 persons in 2012 [16]. However, this number declined to 120 new cases per 100,000 population in 2016 [17]. According to the WHO surveillance data, the estimated TB incidence rate in Sudan declined from 25 per 100,000 population in 2017 to 10 per 100,000 population in 2019 [18]. However, following the COVID‐19 pandemic, the incidence rate jumped to 50 cases per 100,000 population [1] (Figure 2A).

According to the Global TB Report 2025, Sudan is classified in incidence category 5 (10–49 cases per 100,000 population in 2024) and is among the 60 countries that collectively account for 10% of global incident TB cases [19].

The search identified 81 published studies, of which 32 (40%) with relevant data were included in this review. Table 1 summarizes the studies (*n* = 13) that reported the prevalence of TB in Sudan (only studies that reported the prevalence). Overall, very high TB prevalence (> 70%) was reported in the studies that targeted presumptive PTB patients in Kassala State, with > 20% of whom were EPTB patients [25]. This is followed by Gezira State with 26% presumptive PTB prevalence [28]. A multicenter study spanning main cities in Sudan (Gezira, Bahr Al Jebel, Khartoum, Red Sea, Gadarif and Kassala) reported 17% presumptive PTB prevalence; however, this data was generated during the late 1990s [20]. We noted that the prevalence of TB in the Khartoum is relatively lower compared to the Eastern region; 16% of children with symptoms suggestive of TB were confirmed to have the disease, while 5% of peritoneal dialysis (PD) patients were infected with PTB [26].

**Table 1:** A summary of the studies reported TB prevalence and patient characteristics in Sudan.

Study year	State	Patient characteristics	No. TB patients (%)	Reference
Pulmonary and Extra pulmonary TB				
March 1998 to March 1999	Gezira, Bahr Al Jebel, Khartoum, Red Sea, Gadarif and Kassala States	Patients who were identified with respiratory tract symptoms	929/5338 (17.4%)	[20]
2005 to December 2011	Khartoum state	350 PD patients	19/350 (5.4%) were positive for active TB	[21]
2010	Kassala States	Women presenting with various gynecologic symptoms	25/2778 (0.9%) with FGTB	[22]
2011	Kassala States	TB patients	492/670 (73.4%) PTB	[16]
			178/670 (26.6%) EPTB	
January 2011 and June 2012	Kassala States	TB patients	761/985 (77.3%) with PTB	[23]
			224/985 (22.7%) with EPTB	
2011–2013	River Nile State	Overall TB notifications	187/1221 (15.3%) were children, of which 115/187 (61.5) were PTB cases, and 72/187 (38.5%) were EPTB	[24]
2020	Kassala States	Newly diagnosed TB patients	188/251 (74.9%) with PTB	[25]
			63/251 (25.1%) with EPTB	
2016	Khartoum State	Children less than 15 years old suspected of having TB	32/197 (16.2%)	[26]
2017‐2019	Gezira	Presumptive PTB participants	103/300 (34%)	[27]
January 2019 to January 2021	Gazira state	Sputum from suspected patients	127/483 (26.3%)	[28]
Latent TB				
2017	Gazira state	HCW in TB centers	131/367 (35.7%) with LTBI	[29]
November 2015 and April 2016	Gazira state	HHCs of TB patients	43/657 (6.5%) LTBI	[30]
Not mentioned	Not mentioned	98 HHCs of TB patients	27/98 (27.5%) LTBI	[31]

Abbreviations: FGTB, Female genital TB; HCW, Health care workers; HHCs, Household contacts; LTBI, Latent TB infections; PD, peritoneal dialysis.

Data on the prevalence of latent TB infection (LTBI) in Sudan are lacking; nonetheless, two studies from Gezira State reported 35% LTBI among Health care workers (HCW) and 6% among household contacts [29, 30].

### 3.2 Molecular Epidemiology of MTB

Few studies conducted MTB strain typing in Sudan [13, 32, 33, 34]. Spoligotyping is the most used typing method. The CAS1_Delhi/family seems to be the predominant type, accounting for 52% (62/118) of a 2007‐2009 *MTB* complex (MTBC) strains collection and 49% (114/232) in Khartoum State and in Central and Eastern Sudan, respectively [13, 34]. This is followed by the H3/family (16%) strains, and the Latin American Mediterranean3 LAM3/family, LAM10_CAM, T2/T1, and two strains each of the Beijing and S lineage [34]. However, a more diverse population was noted during 2011‐2012 in Kassala and Gezira states where the Euro‐American, Central Asian, and Indo‐Oceanic Lineages in the following clades (out of 92 MTBC strains): SIT54 (Manu2) (64.8%), CAS (22.7%), EAI (4.5), LAM2 (2.3%), H3 (1.1%), T (1.1%), T3‐ETH (1.1%), T5‐RUS1 (1.1%) were reported [33].

Sub‐lineage (L) classification (using a single‐nucleotide polymorphisms SNP barcode nomenclature) conducted on genome sequences of 166 MTBC strains, reported that L3 (Delhi/CAS) is the predominant strain accounting for 73.5% (122/166), followed by L4 (Euro‐American) strains 23.5% (39/166), L1 2.4% (4/166) and L2 0.6% (1/166) [32].

### 3.3 TB Diagnosis

The TB control program in Sudan is based on a passive and voluntary case approach, and compliance with treatment is optional [35]. Despite the advances in TB diagnostic technologies worldwide, particularly the development of molecular testing led by the GeneXpert platform (Cepheid, USA), and more recently the Truenat platform (Molbio, India), TB diagnosis in Sudan still mainly relies on clinical symptoms, Ziehl‐Neelsen (ZN) microscopy, solid MTB culture (Löwenstein–Jensen (LJ) medium) and tuberculin skin test (TST). TB diagnosis is only done at specialized referral laboratories where most presumptive PTB patients are sent for sample collection and testing. ZN staining is routinely used in all TB diagnostic centers across the country, whereas the Auramine Fluorochrome method is restricted for use in the National Reference Laboratory (NRL) [2]. Therefore, the local laboratories have limited access to mycobacterial culture and drug susceptibility testing (DST) or DNA amplification‐based techniques [32]. This adds complications due to the delayed diagnosis and treatment initiation, contributing to further spread of the disease.

Data on the number of TB notifications was only available through the WHO reports. In 2016, a total of 21,084 TB notifications were reported, of which 76% were pulmonary TB (PTB). Among all reported cases, 46% were bacteriologically confirmed using available diagnostic methods (including smear microscopy, culture, and/or molecular testing), while the remaining cases were clinically diagnosed in accordance with WHO TB case definitions (WHO, 2017). In 2017, 21 Gene Xpert platforms were installed in 8 laboratories across the country [36]. In 2018, only up to 24% of the notifications were tested using a WHO‐recommended rapid test [15]. The overall number of notifications dropped in 2018 to 20,638 cases, with 73% being PTB, but 50% of the cases were bacteriologically confirmed, indicating the improvements following the implementation of rapid molecular testing at central laboratories [1, 15].

Generally, TB diagnosis in children is more difficult due to insufficient specimen material and the scarcity of bacilli in specimens. It is established that nucleic acid amplification techniques are the most sensitive and specific for the fast identification of MTB in gastric aspirates and sputum from children who are unable to expectorate a good‐quality sputum sample or who are diagnosed as negative using conventional diagnostic methods [2]. However, in Sudan, laboratories are still relying on microscopy as the primary diagnostic test, leading to missing TB cases amongst children due to the paucibacillary nature of the disease in this age group.

A comparative study on the challenges of TB diagnosis in Sudanese children investigated the performance of TST, microscopy (ZN and Auramine), and conventional PCR using sputum culture as a reference standard. The study concluded that despite the TST achieving 100% sensitivity based on the reference standard, culture on LJ medium was more specific because it confirms *true infection* by isolating live *M. tuberculosis*; the performance of the different methods was 100% sensitivity for TST, 43.8% for ZN, 56.3% for Auramine, and 100% for PCR [26].

A study compared the performance of ZN sputum smear done at local laboratories in Sudan and the National Reference Laboratory (NRL) in Germany. They showed suboptimal concordance; one‐third of the samples (*n* = 123, 32.1%) that tested smear positive in Sudan were categorized as smear negative at the NRL in Germany, and 10% of samples classified as smear positive in Sudan did not have any evidence of mycobacteria using culture or PCR as the reference standard in Germany [37]. This is concerning, as the country is relying on smear microscopy as the main diagnostic method, as in many low‐income countries. In settings where smear microscopy cannot be avoided, adequate training, regular supervision, and national external quality assurance schemes are critical to reduce reading and staining errors in settings. Additionally, the use of highly specific molecular diagnostic assays to differentiate MTB from nontuberculous mycobacteria (NTMs) is crucial. Where available, these molecular tests should ideally replace smear microscopy, as they offer superior sensitivity and specificity and can substantially improve diagnostic accuracy in these settings.

Data on the diagnosis of extrapulmonary TB (EPTB) in Sudan are scarce. However, one study reported that abdominal TB can be diagnosed when there is strong clinical suspicion together with one or more of the following: caseous necrosis or granulomas on tissue biopsy; a positive polymerase chain reaction (PCR) test for *M. tuberculosis*; detection of acid‐fast bacilli by Ziehl–Neelsen staining or culture; a strongly positive tuberculin skin test (> 15 mm induration) in a non‑BCG‑vaccinated patient; suggestive ascitic or pleural fluid findings accompanied by radiological features consistent with TB; or a clear clinical response to empirical antituberculous therapy [21].

The health system in Sudan is fragile and has been affected by poor management and low public expenditure. This has a drastic impact on managing infectious diseases such as TB, which requires timely diagnosis and treatment initiation. Sudan is still lagging in terms of decentralizing TB diagnosis. There is a need for scaling up the use of molecular testing using Xpert MTB/Ultra and Line probe assay (LPA) and introducing biomarker‐based point‐of‐care tests such as C‐reactive protein, which proved to be superior to symptom screening [38].

### 3.4 Antimicrobial Resistance Profiles and Resistances Genes

**Figure 3:**
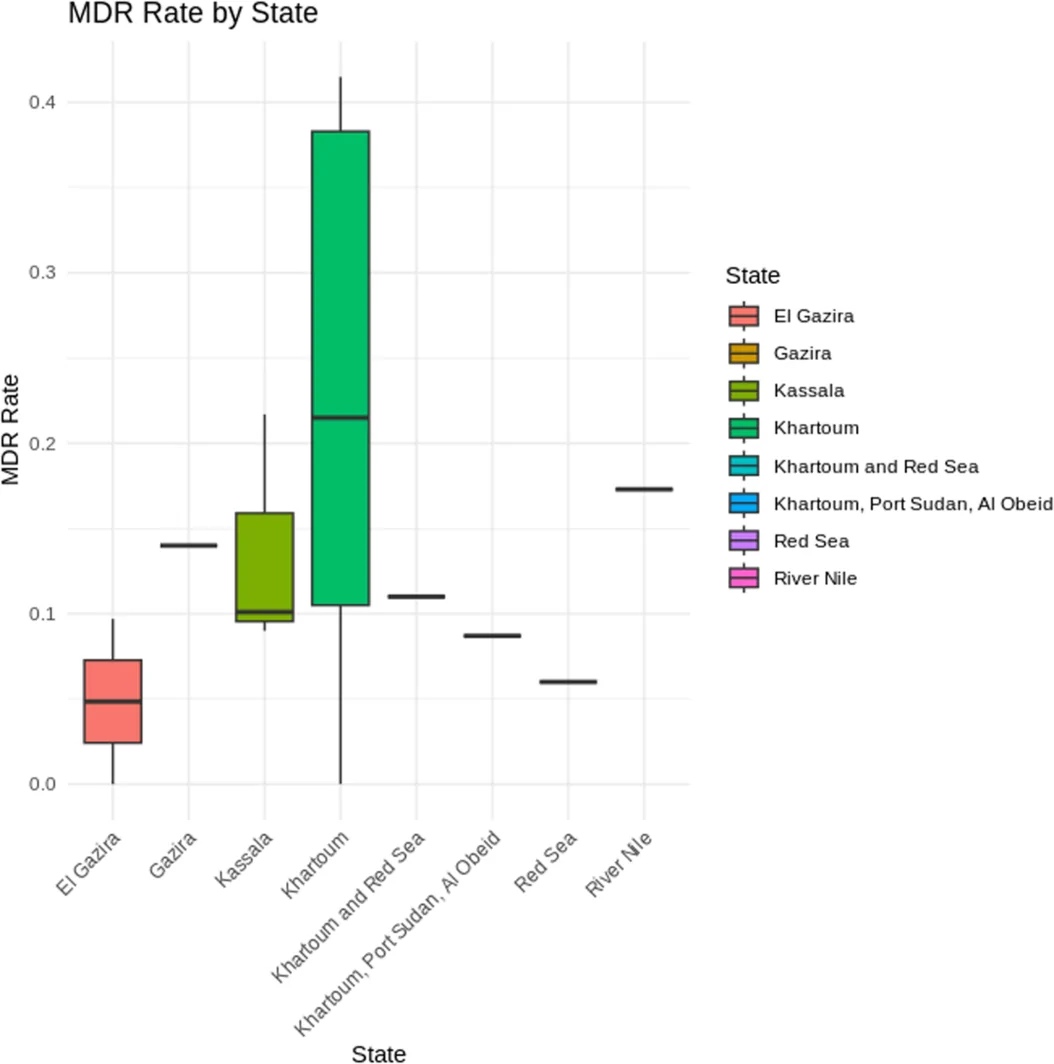
MDR rate according to different Sudanese states.

Table 2 summarizes the 19 studies that reported drug resistance and the associated genes. Based on the studies done in a single state, the highest number of MDR cases was observed in Khartoum State, 76/183 (41.5), as reported by eleven studies [13, 39, 40, 43, 44, 46, 47, 48, 51, 52, 53], followed by Kassala State 5/23 (21.7%) [32, 41, 54]. Whereas River Nile, Gaziera, and Red Sea states reached 9/52 (17.3%), 21/150 (14%), and 6/100 (6%), respectively [27, 28, 42, 49, 50], (Figures 2B and 3).

**Table 2:** A summary of the studies reported TB drug resistance and the associated genes in Sudan.

Study year	State	Patient characteristics	No. patients	Drugs tested (% resistance)	MDR n/N (%)	Identified resistance genes	Reference
September 1998 to October 1999	Khartoum State	Patients with positive smears	50	NR	2/50 (4)	*katG, rpoB, rpsL, rrs, embB*	[39]
July 2001 to December 2001	Khartoum and Gazira and camps for displaced people	PTB patients	144	INH (11.8)* (59)#, RIF (3.2)* (57)#, STM (35.4)* (69)# and EMB (41)#	31/144 (21.5)	NR	[40]
May to December 2005	Khartoum state and red sea state	MTB from new and previous PTB cases	235	INH (14), RIF (22), STM (32) and EMB (22)	26/235 (11) (5)* (24)#	NR	[13]
January 2006 to June 2008	Kassala State	MTB isolates from PTB patients	53	INH (NR), RIF (56), STM (48) and EMB (NR)	5/23 (21.7)	*rpoB*	[41]
March 2006 to March 2007	Red Sea state,	MTB isolates identified from newly identified TB cases	100	RIF (8), INH (13), STM (34), PZA (1) EMB (12)	6/100 (6)	*rpoB* 531 (71.4%) and *rpoB* 526 (28.6%) for RIF, *katG* 315 (90%) and *mabA‐inhA*−15, *embB, rpsL*	[42]
2007 and 2009	Khartoum state	RIF resistant‐TB	49	RIF (100), INH (100), STM (84), EMB (77)	NR	*rpoB*	[43]
July 2009 to July 2010	Khartoum state	smear positive retreatment TB cases	141	RIF (1.4), INH (2), STM (12) and EMB (1.4)	54/141 (38.3)	NR	[44]
November 2011 to October 2012	Kassala and Geddarif	new and previous PTB cases	109	INH, RIF, STM and EMB	11/109 (10.1). 4/11(36.3)* 7/11 (63.6)#	NR	[45]
December 2011 to March 2015	Khartoum state	PTB patients	126	RIF (48), INH (52)	42/126 (33.3) 4/42 (9.5)* 37/42 (88.2)#, 1/42 (2.3) unknown case	*rpoB, KatG, InhA*	[47]
February 2015 to July 2015	Khartoum state	Drug resistant TB patients	70	RIF (31), INH (41), PZA (47)	29/70 (41.4)	*rpoB, katG* and *pncA*	[32]
2014 and 2016	Kassala, Red Sea, and El‐Gadarif	Smear positive PTB patients	166	INH (10), RIF (10), PZA (0.6), STM (20) and EMB (0.6)	15/166 (9)	*rspL, gidB, rrs, rpoB, katG, fabG1*‐*inhA, embB*	[26]
August 2017 to January 2018	Khartoum State	PTB patients	183	NR	76/183 (41.5)	NR	[48]
during 2017 to 2019	El Gazira State	presumptive PTB patients	103	NR	10/103 (9.7)	*rpoB*	[27]
September 2020 to June 2021	Gazira State	TB patients	150	NR	21/150 (14)	NR	[49]
July 2019 to December 2019	River Nile State	PTB patients	52	NR	9/52 (17.3)	NR	[50]
January 2019 to January 2021	El Gazira State	PTB patients	127	RIF (7)	NR	*rpoB*	[28]
Not mentioned	Khartoum State	MTB isolates from PTB patients	200	INH (17.5), RIF (15.5), STM (21.5) and EMB (12)	21/200 (10.5)	NR	[51]
Not mentioned	Khartoum, Port Sudan, and Al Obeid	MTB isolates from PTB patients	149	RIF (21), IHN (18), STM (33), EMB (22)	13/149 (8.7%), 6/13 (46.1)*, 5/13 (38.5)#	NR	[52]
Not mentioned	Khartoum State	PTB patients	75	INH (28), RIF (29), STM (56) and EMB (20)	15/75 (20) (5.3)* (14.7)#	*rpoB, KatG, InhA, rrs*	[53]

* New cases,

^#^ Previously treated.

Abbreviations: EMB, Ethambutol; INH, isoniazid; MTB, *Mycobacterium tuberculosis*; NR, not reported; Not mentioned, the date not mentioned; PTB, Pulmonary TB disease; PZA, pyrazinamide; RIF, Rifampicin; STM, streptomycin; TB, Tuberculosis disease.

**Figure 4:**
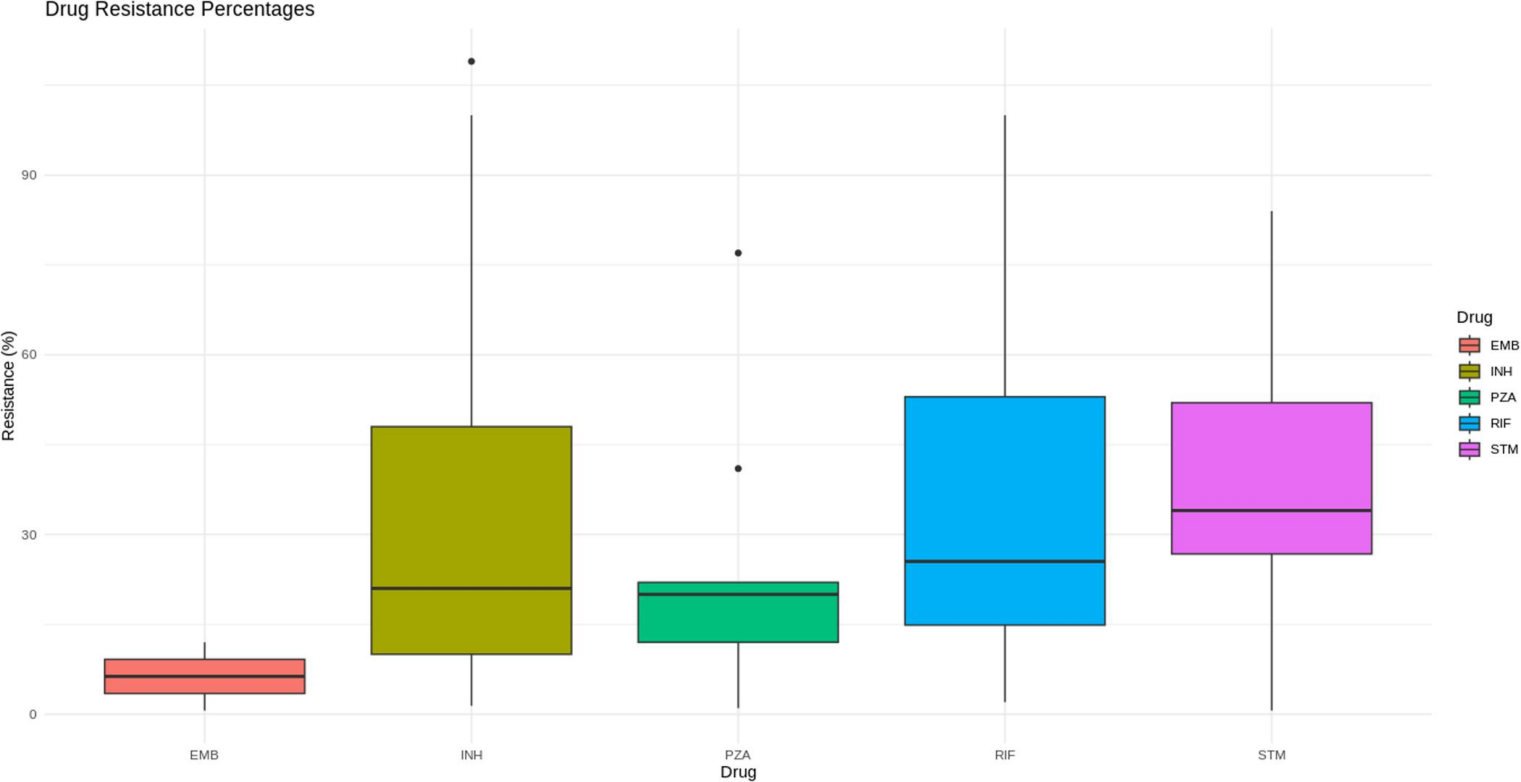
Drug resistance percentage.

**Figure 5:**
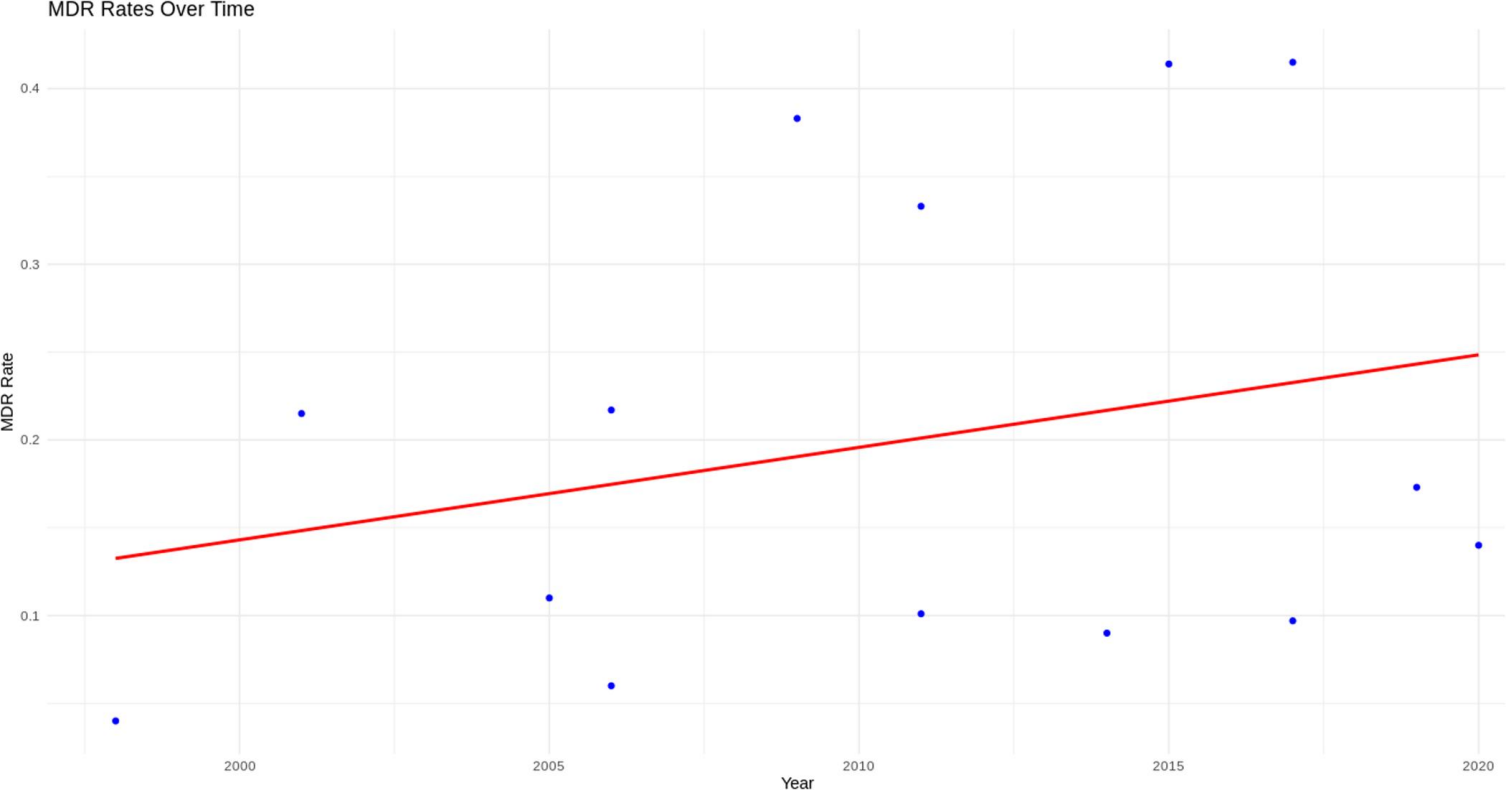
The variability in MDR trends over time, showing increasing trends over time.

The mean resistance levels varied across drugs, with INH (34.92%) and STM (39.74%) showing the highest means, while EMB had the lowest (6.3%) (Figure 4). However, statistical analysis revealed no significant differences in resistance levels between the drugs (ANOVA, *p* = 0.50). Tukey's HSD post‐hoc test confirmed no significant pairwise differences between drugs, as all adjusted *p*‐values were > 0.5 and confidence intervals included zero. The results show only a small fraction of the variability in MDR trends over time (*R*‐squared value was 0.071) (Figure 5) and significant differences in MDR rates between states (Figure 3).

**Figure 6:**
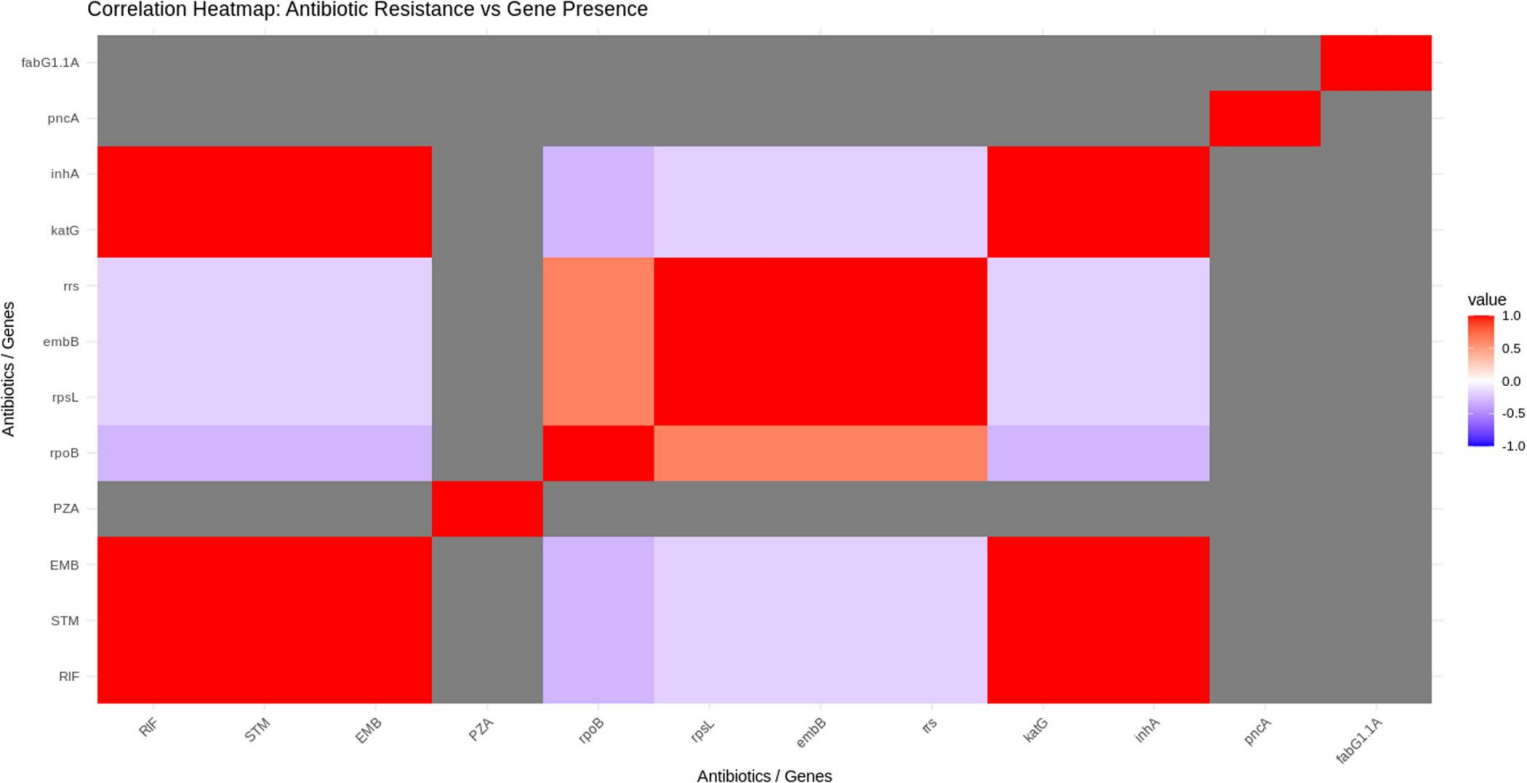
Correlation heatmap of antibiotic resistance and presence of gene mutations.

Studies conducted in Sudan reported mutations in the *rpoB, katG, inhA, pncA, gidB, rrs, rspL, fabG1‐inhA, embB* genes. RIF resistance, mediated by the *rpoB* gene, was the most predominant [28, 32, 39, 41, 42, 43, 46, 47]. Logistic regression analysis predicted resistance to RIF based on gene mutation presence and revealed that the *katG* gene appears to have a potentially significant impact on RIF resistance, while other genes like *rpoB*, *rpsL*, and *embB* seem to have little to no effect (Figure 6).

The drug resistance data reported in this review does not necessarily reflect the real distribution of drug resistance at a national level. According to the WHO, Sudan suffers from a fragmented and non‐aggregated Health Information system and weak coordination across all health sectors, leading to the difficulty in tracking pivotal information on TB notifications and drug resistance [36].

### 3.5 Treatment Regimens

The first‐line drugs used to treat TB in Sudan are isoniazid (INH), rifampicin (RIF), pyrazinamide (PZA), ethambutol (EMB) and streptomycin (STM). While, kanamycin, capreomycin, ethionamide, cycloserine, ofloxacin and ciprofloxacin are used as second‐line drugs. A 6‐month treatment course is usually sufficient in uncomplicated cases of PTB. The retreatment regimen of multi‐drug‐resistant patients is augmented by using second‐line drugs, such as fluoroquinolone or injectable streptomycin until drug sensitivity data are available [21]. In 2016, the WHO reported a treatment success rate of 78% among new and relapse cases and a 21% success rate for MDR/RR TB cases [55].

A study conducted in Kassala State, one of the states with the highest TB burden, found that the most important predictor of treatment default was discontinuation of therapy after patients began to feel better and mistakenly believed they were cured, particularly at the start of the continuation phase. Non‑adherence to treatment is influenced by several factors, including poor patient knowledge and awareness of TB and its treatment, low levels of education and income, as well as the patient's age and place of residence [17].

**Figure 7:**
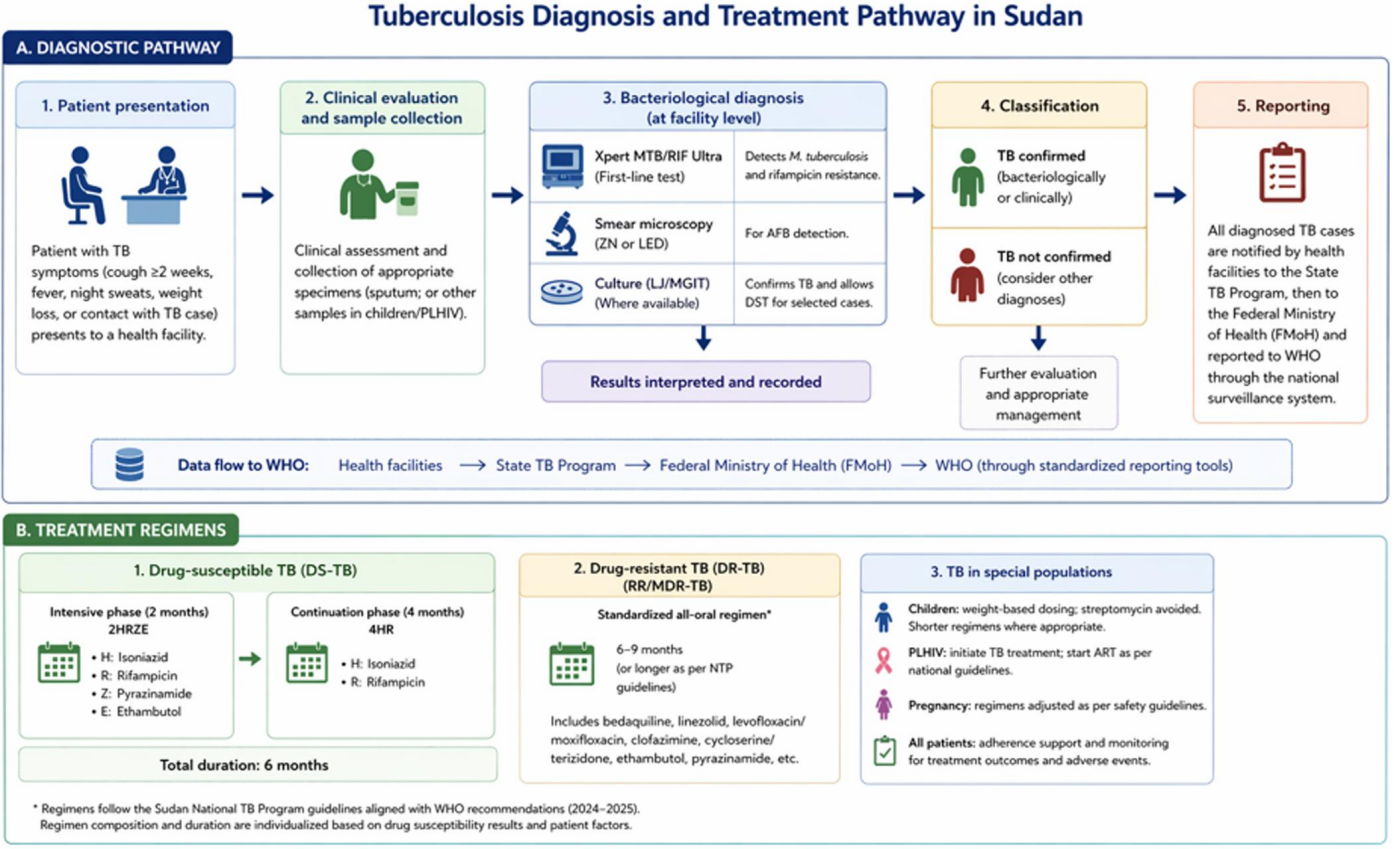
Flowchart of TB diagnosis and treatment regimens in Sudan. The diagram illustrates the stepwise pathway for TB management in Sudan, starting from patient presentation and clinical evaluation, followed by bacteriological diagnosis using Xpert MTB/RIF Ultra, smear microscopy, and culture methods. Confirmed cases are classified and reported through the national surveillance system from health facilities to the State TB Program, the Federal Ministry of Health, and finally to WHO. The lower section outlines standard treatment regimens for drug‐susceptible TB, drug‐resistant TB, and special populations according to national TB program guidelines aligned with WHO recommendations. AFB, Acid‐fast bacilli; ART, Antiretroviral therapy; DST, Drug susceptibility testing; FMoH, Federal Ministry of Health; LED, Light‐emitting diode; LJ, Lowenstein–Jensen medium; MGIT, Mycobacterial Growth Indicator Tube; NTP, National Tuberculosis Program; PLHIV, People living with HIV; RR/MDR‐TB, Rifampicin‐resistant/multidrug‐resistant tuberculosis; TB, Tuberculosis; WHO, World Health Organization; ZN, Ziehl–Neelsen staining.

Patients with extrapulmonary TB (EPTB), including those with comorbid chronic kidney disease or receiving dialysis, were treated with a standard TB regimen consisting of a two‑month intensive phase of isoniazid, rifampicin, pyrazinamide, and ethambutol, followed by a four‑month continuation phase with isoniazid and rifampicin. Dose adjustments for ethambutol and pyrazinamide were made where indicated. An extended treatment duration of up to 12 months was used in patients with TB of the bones and joints and in those with tuberculous meningitis. In cases of TB meningitis, ethambutol was replaced with injectable streptomycin. See Figure 7 for TB diagnosis and the treatment regimens in Sudan.

### 3.6 HIV‐TB Co‐Infection

According to the UNAIDS, the prevalence of HIV amongst Sudanese people aged 15–49 years is 0.1% (0.1–0.2) [56], and over the years, the range of HIV prevalence in new and relapse TB cases remained between 0% and 4.9% [1, 7, 15, 18, 55].

Studies included in this review emphasized the lack of HIV data; for example, only 13% (109/858) of TB patients in Kassala state were tested for HIV [57], and in River Nile state, only 22% of the 1221 TB patients agreed to be tested [58]. The very low numbers of people agreeing to be tested for HIV in these studies are mainly driven by the stigma associated with HIV in our communities. Nonetheless, a multistate study conducted from March 1998 to March 2000 in eight states namely, Gezira, Bahr El Jabal, Khartoum, Red Sea, Gadarif, North Kordofan, West Bahr Elgazal, and Kassala states, including internally displaced persons from five camps (four in Khartoum and one in North Kordofan) reported a 4% (73/1797) HIV coinfection rate amongst TB patients [59].

Kassala State showed a high co‐infection rate ranging from 7.3% (24/389) to 21.6% (24/111) [25, 57, 60, 61, 62]. While the co‐infection rate in Khartoum State ranged from 4.3% (12/281) to 11.3% (9/80) [63, 64, 65, 66], and 4% (10/269) in River Nile State [58], and from 4% (5/127) to 8% (12/150) in Gazira State [28, 49]. We believe that the issue of stigma has a significant impact on HIV reporting; therefore, the prevalence of TB‐HIV coinfection in Sudan is likely under‐reported and underestimated.

### 3.7 TB Drivers in Sudan

In 2022, the WHO reported that TB cases in Sudan are attributable to four risk factors, with undernourishment being in the lead. This is followed by Diabetes, alcohol use disorders, and HIV [67]. This TB‐undernutrition syndemic is common in almost all high TB burden settings with high poverty rates. In Sudan, approximately 47% of the population live below the poverty line [68].

This underscores the need for governments and National TB Programmes to explore complementary interventions, such as the provision of nutritional support to patients with TB, particularly those at high risk of infection, as a strategy to reduce TB incidence.

The effectiveness of this approach has been demonstrated in a clinical trial conducted in India, where nutritional supplementation among exposed household contacts was associated with a 39%–48% reduction in incident TB compared with those who did not receive nutritional support [69]. In the Sudanese context, such an intervention could have a substantial impact, particularly in regions affected by ongoing conflict, famine, and drought, such as Darfur in the western part of the country.

### TB Research Funding

The biggest international funders for TB prevention, diagnosis and treatment from individual countries are the United States of America (USA), followed by the United Kingdom (UK) [70]. The impact of the sanctions imposed by the USA on TB research is substantial, creating significant barriers for international organizations and researchers seeking to allocate funds to support TB‐related initiatives in the country.

Despite the notable 3.9% increase in the global total spending on TB per year in low‐income and middle‐income countries from $5.7 billion in the year 2000 to $10.9 billion in 2017 [71], in Sudan, TB funding decreased substantially over the past 5–10 years (2017–2025) [67]. In 2017, the country received US$15 million, 83.3% of which was from international funders, while only 17% was from domestic funders. This reduced even further to only US$ 5 million in 2021, with only 19% being domestic funding [67]. The economic instability within the country limits the government's ability to allocate substantial resources to healthcare, including research initiatives. Also, political uncertainties and ongoing conflicts create an environment that hampers the effective implementation of research programs. Additionally, the lack of a well‐established research ecosystem and limited international investments make it challenging to attract and retain skilled researchers, hindering the overall progress in TB research.

### 3.8 TB Coinfections and Risk Factors

Although not the focus of the review, we included the sections below for the purpose of reflecting on the research done on coinfection and risk factors, mainly due to the scarcity of data on TB and its risk factors in Sudan.

### 3.9 TB Co‐Infection

Due to the high morbidity rate amongst TB patients, they are at a higher risk of being infected with other pathogens. Few studies reported data on the HBV/HCV‐TB coinfection in Sudan and these were limited to Eastern and Central regions. Serological tests were used to confirm the seroprevalences, which ranged from 3.1% (3/98) for anti‐HCV, 17.3% (17/98) for HBsAg, and 3.1% (3/98) were coinfected with both HBV and HCV and TB in Kassala state [72]. Other studies reported seroprevelance ranged from 5.3% (1/19) to 9.5% (19/200) for HBV, 3.5% (7/200) for HCV and 1% (2/200) were coinfected with HBV‐HCV and TB, respectively, in Khartoum state [21, 73].

Despite the limited data on coinfection with viral pathogens in Sudan, our data suggest that there are un negligible numbers amongst TB patients which might lead to a complicated treatment outcome. Another study in Khartoum state reported that 17.2% (11/64) of TB patients were infected with *Toxoplasma gondii* [74]. A multipathogen detection PCR panel would be an ideal approach to identify the respiratory pathogens coexisting with TB, and in some cases, causing disease when the patient is deemed TB negative. However, all existing technologies are not yet tailored for point‐of‐care testing in resource‐limited settings [75].

Sudan is a Leishmania endemic country. Using leishmanin skin test (LST) and a tuberculin test, a study conducted in Eastern Sudan reported a 20% (77/382) coinfection rate of TB with *Leishmania donov*ani [76].

### 3.10 TB Risk Factors

Type 2 Diabetes Mellitus (T2DM) is the dominant type in Sudan and is considered the second attributable risk factor for active TB in the country [67]. A study conducted in Khartoum State in 2022 investigated the association between T2DM and its duration and PTB among Sudanese patients, as well as the association between the levels of hemoglobin A1c (HbA1c) in diabetic patients and development of pulmonary TB. The study reported consistent evidence for an increased risk of TB among patients with uncontrolled DM (high‑level HbA1c) [77].

A study conducted in Khartoum State between 2014 and 2015, which investigated the occurrence of TB in maintenance the study reported a TB incidence rate of 1.4% amongst HD patients [78].

Vitamin D deficiency is considered a risk factor for the development of TB. A single study that was conducted in the White Nile State confirmed that vitamin D levels were significantly lower in PTB patient a oppose to those with no TB [79].

### 3.11 Challenges Associated With TB in Sudan

Combating TB in Sudan presents several challenges. One significant obstacle is the limited healthcare infrastructure exacerbated by economic instability and political uncertainties. Insufficient resources, both financial and technological, impede effective TB diagnosis, treatment, and prevention measures. The ongoing conflicts in certain regions of Sudan further strain healthcare delivery, hindering access to affected populations. Sociocultural factors, including stigma and misconceptions surrounding TB, contribute to delayed diagnosis and treatment initiation [11]. A study in El Gazira State was conducted to evaluate the prevalence of TB stigma and reported that supporting both TB patients and communities by increasing their knowledge through good education programs could effectively contribute to the effort of controlling TB in the state [80]. Also, in Kassala State, where the study was conducted to evaluate the stigma towards TB patients, they noted the high stigmatizing attitude towards TB patients, which influences the effectiveness of TB control [81]. Also, this stigma leads to isolation from families and friends, loss of employment, and exclusion from community activities [82, 83].

Additionally, the country faces challenges in sustaining healthcare programs due to the competing health priorities and varying levels of international assistance. Addressing these multifaceted challenges requires a comprehensive and sustained effort, integrating healthcare infrastructure development, public awareness campaigns, and international collaboration.

## 4 Conclusions and Recommendations

This review demonstrates substantial gaps in TB data across Sudan, particularly in the western regions, and highlights marked geographic disparities in disease burden. One of the most concerning findings is the notably high rate of multidrug‐resistant TB (MDR‐TB) in Khartoum, which reflects long‐standing weaknesses in diagnostic coverage, treatment adherence, and surveillance systems. These challenges have been further compounded by recent conflict‐related disruptions, which have strained health services, hindered access to care, and contributed to delays in diagnosis and treatment interruption, conditions that facilitate the emergence and spread of drug‐resistant strains.

Strengthening the country's response requires a multifaceted approach. An integrated health information system (HIS) is essential to generate accurate, timely, and representative data for effective planning and research, including molecular epidemiology studies. Decentralizing TB services and scaling up point‐of‐care and near‐point‐of‐care molecular diagnostics are critical steps to bring testing closer to communities, enabling earlier case detection, continuous monitoring, and rapid intervention.

Given the rising MDR‐TB burden, rapid and reliable drug‐resistance testing must be prioritized to prevent misuse of first‐line TB drugs and ensure timely initiation of appropriate second‐line regimens. Optimizing MDR‐TB management with effective, less toxic treatment options should remain central to national TB strategies. Additionally, nationwide awareness campaigns are needed to combat stigma and encourage health‐seeking behavior, while targeted nutritional support is vital, as malnutrition remains one of the major drivers of TB vulnerability and poor treatment outcomes in Sudan.

Addressing the persistent challenges in TB control and research funding will require coordinated national and international efforts to rebuild health infrastructure, stabilize supply chains, support health‐care workers, and promote sustained investment in TB research and programmatic capacity. Only through such comprehensive and resilient strategies can Sudan effectively curb TB transmission and reduce the burden of drug‐resistant disease.

## Author Contributions

**Mohammad Azhar Kamal:** validation, writing – reviewing and editing. **Hisham N. Altayb:** validation, writing – reviewing and editing, data analysis. **Shima M. Abdulgader:** conceptualization, methodology, data curation, writing – original draft preparation and supervision. **Ehssan Moglad:** conceptualization, methodology, data curation, writing – original draft preparation, visualization, investigation, funding acquisition and supervision. All authors read and approved the final version of the manuscript. All authors have read and approved the final version of the manuscript. Ehssan Moglad had full access to all of the data in this study and takes complete responsibility for the integrity of the data and the accuracy of the data analysis.

## Conflicts of Interest

The authors declare no conflicts of interest.

## 1 Transparency Statement

Ehssan Moglad affirms that this manuscript is an honest, accurate, and transparent account of the study being reported; that no important aspects of the study have been omitted; and that any discrepancies from the study as planned (and, if relevant, registered) have been explained.

## Data Availability

The data that support the findings of this study are available from the corresponding author upon reasonable request.
